# Fresh embryos versus freeze-all embryos - transfer strategies: Nuances of a meta-analysis

**DOI:** 10.5935/1518-0557.20170048

**Published:** 2017

**Authors:** Felipe C Dieamant, Claudia G Petersen, Ana L Mauri, V. Comar, Mariana Mattila, Laura D Vagnini, Adriana Renzi, Bruna Petersen, Andreia Nicoletti, João Batista A Oliveira, Ricardo LR Baruffi, Jose G Franco Jr

**Affiliations:** 1 Center for Human Reproduction Prof. Franco Jr., Ribeirão Preto, Brazil; 2 Paulista Center for Diagnosis, Research and Training, Ribeirão Preto, SP, Brazil

**Keywords:** embryo transfer, ovarian stimulation, oocytes, oocyte retrieval, ICSI, meta-analysis

## Abstract

**Objective:**

The present meta-analysis aimed to evaluate whether the freeze-all strategy (Freeze/All-ET) could bring about improvements in the clinical assisted reproductive technique (ART) outcomes when compared with the fresh embryo transfer strategy (Fresh-ET) in patients undergoing an ART cycle in accordance with the mean number of oocytes collected.

**Methods:**

A systematic review based on electronic searches in databases (PubMed, EMBASE, Web of Science, SCOPUS, and Cochrane Central Register of Controlled Trials) was carried out to identify randomized controlled trails (RCTs) comparing ART outcomes between fresh-embryo transfers versus elective frozen-embryo transfers up to February of 2017. Four reviewers independently evaluated abstracts, validity assessment and data extraction. Odds Ratio (OR) values with a 95% confidence interval (CI), and heterogeneity were evaluated.

**Results:**

Five RCTs were included as targets for data extraction and meta-analysis purposes. The results of this meta-analysis were divided into two parts (Freeze/All-ET versus Fresh-ET): Part I- All trials in which the mean number of collected oocytes was >12 and <21 for ongoing pregnancy rate (OR=1.24; 95%CI=1.06-1.44), clinical pregnancy rate (OR=1.19; 95%CI=0.98-1.43), live birth rate (OR= 1.39; 95%CI=0.99-1.95), and miscarriage rate (OR=0.68; 95%CI=0.46-1.00); Part II- Three studies where the mean number of oocytes retrieved was >12 and <15 for ongoing pregnancy rate (OR=1.17; 95%CI=1.00-1.38), clinical pregnancy rate (OR=1.34; 95%CI=0.79-2.28), live birth rate (OR= 1.24; 95%CI=1.00-1.55), and miscarriage rate (RR=0.68; 95%CI=0.46-1.02).

**Conclusions:**

The freeze-all strategy could be favorable when high numbers of oocytes are collected, signaling an association between higher ovarian stimulation and consequent impairment of endometrial receptivity. However, when the mean number of oocytes collected is <15, the freeze-all strategy does not appear to be advantageous.

## INTRODUCTION

The Freeze-all strategy (Freeze/All-ET), which consists of the cryopreservation of all embryos from an assisted reproductive technique (ART) cycle, and delayed embryo transfer in a natural cycle or a programmed hormone replacement cycle to prepare the endometrium, which is considered the preferred way to avoid potential deleterious effects of controlled ovarian stimulation (COS) during fresh-embryo transfer (Fresh-ET) on endometrium receptivity, and consequently on embryonic implantation (Silverberg *et al.*, 1994; Shapiro *et al.*, 2008). COS is associated with negative effects on endometrial receptivity during ART cycles, probably due to high levels of estrogen (E) and progesterone (P) during the follicular phase compared to natural cycles (Kolibianakis *et al.*, 2002; Bosch *et al.*, 2003; Venetis *et al.*, 2013; Huang *et al.*, 2015). Because of subtle elevations of P during COS, there could be a consequent asynchrony between the endometrium and the transferred embryos; probably the endometrial development should be at an advanced stage at the moment of embryonic implantation (Nikas, 1999; Wong *et al.*, 2014). Therefore, it's known that the best results in ART, considering pregnancy rates, are found in oocyte donation cycles and cycles using frozen-thawed embryos transfer (FET) (Murata *et al.*, 2005; Richter *et al*., 2006; Shapiro *et al.*, 2009; Kansal Kalra *et al.*, 2011). A plausible explanation for this is the fact that the endometrium is artificially primed, without COS and supraphysiological hormonal levels at the time of the embryo transfer (Melo *et al.*, 2006; Venetis *et al.*, 2013). In addition, it has been reported that patients with high ovarian reserve, e.g. high-risk of ovarian hyperstimulation syndrome (OHSS), and polycystic ovarian syndrome (PCOS) patients, could benefit from the Freeze/All-ET (Griesinger *et al.*, 2007; Griesinger *et al.*, 2011).

The aim of the present systematic review and meta-analysis is to evaluate whether Freeze/All-ET could bring about improvements in the clinical ART outcomes when compared with Fresh-ET in patients undergoing the ART cycle, in accordance with the mean number of oocytes collected.

## MATERIALS AND METHODS

### Identification of studies

We ran a systematic review based on electronic searches in the following databases (PubMed, EMBASE, Web of Science, SCOPUS, and Cochrane Central Register of Controlled Trials), up to February of 2017, to identify randomized controlled trials (RCTs) comparing ART outcomes of Freeze/All-ET versus Fresh-ET. The search was restricted to papers published in English. The following medical subject headings and text words were used: "IVF", "ICSI", "freeze-all", "frozen-thawed embryos", "frozen-embryo transfer", "fresh-embryo transfer", "poor-responder", "normal-responder", "high-responder", "clinical outcomes", "oocytes collected", and "randomized study". The main inclusion criterion was a randomized controlled trial (RCT).

### Criteria for including studies in this meta-analysis

All available published and ongoing randomized controlled trials comparing clinical outcomes between patients undergoing IVF/ICSI cycles with Freeze/All-ET or Fresh-ET were included. All trials provided data on IVF cycles, including number of oocytes retrieved.

### Outcome measures

The primary outcome measure for this meta-analysis was the ongoing pregnancy rates (per woman, randomized). Secondary outcomes included clinical pregnancy rates (per patient randomized) and miscarriage rates (from clinical pregnancy). Clinical pregnancy was defined as the presence of a gestational sac in the uterine cavity (with or without a heartbeat) at 6/7 gestation week, detected by ultrasonography. Ongoing pregnancy was defined as the presence of a fetus with heart motion at 10 to 12 weeks of gestation. Miscarriage was considered any pregnancy - clinical pregnancy - that did not achieve ongoing pregnancy status. In addition, live birth rates defined as the delivery of a live-born infant after 25 weeks of gestation was included as secondary outcomes.

### Validity assessment and data extraction

Each trial was assessed independently by four reviewers (FCD, JBAO, RLRB and JGF), and ranked for its methodological rigor and its potential for the introduction of biases. Originally reported characteristics, including a method for randomization, the presence of a power calculation, the unit of analysis used, and the presence or absence of examiner blinding were analyzed. Missing data were obtained from the authors.

### Statistical analysis

Five RCTs were included as targets for data extraction and meta-analysis. The data was combined for meta-analysis using the Stats-Direct statistical software. Dichotomous data was expressed as Odds Ratio (OR) with a 95% confidence interval (CI). The measure of heterogeneity was evaluated using Cochran's Q and I^2^. The heterogeneity was considered high when I^2^≥50%. The study data was combined using a fixed-effects model when the heterogeneity among the trials was considered low or statistically insignificant (I ^2^ was <50%). However, the random-effects model was employed when the heterogeneity was considered substantial (I^2^≥50%), and when I^2^ was not applicable (NA). *P*-values<0.05 were considered statistically significant.

The present meta-analysis was reported following the Preferred Reporting Item for Systematic Reviews and Meta-analyses (PRISMA) statement (S1 File).

## RESULTS

### Study selection and characteristics

**Figure 1.**
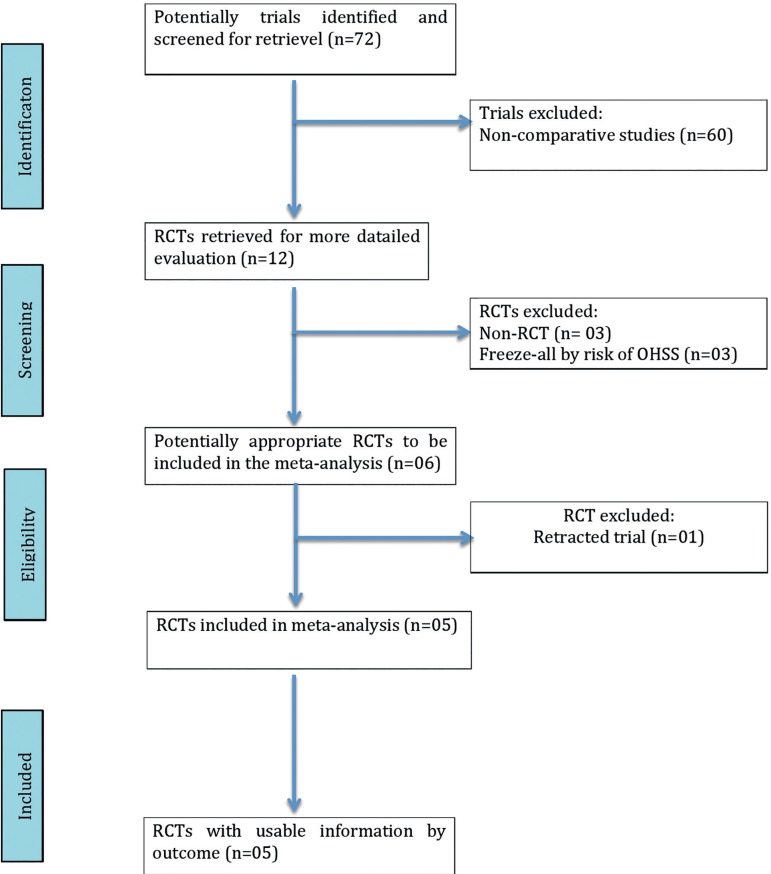
QUOROM statement flow diagram illustrating the selection of trials included in this meta-analysis.

Among the 72 potentially relevant studies found, a total of five trials fulfilled the inclusion criteria (Shapiro *et al.*, 2011a; Shapiro *et al.*, 2011b; Chen *et al.*, 2016; Vuong *et al.*, 2016; Coates *et al.*, 2017). A flow diagram of the selection process is depicted in Figure 1. From the studies included, 2,728 patients were enrolled; 1,358 in the Freeze/All-ET group and 1,370 in the Fresh-ET group. The sample sizes of the included trials ranged between 60 and 762 women. The main characteristics and description of the five RCTs included in this meta-analysis are shown on Table 1 and the literature-exclusion procedures are available in Figure 1.

**Table 1:** Characteristics of the studies included

Study (RCTs)	Women (FET/Fresh-ET)	Age, years (FET/Fresh-ET)	Mean number of oocytes retrieved (FET/Fresh-ET)	Day of embryo transfer	Outcomes measured
Shapiro 2011a	122 (60/62)	30.6/31.4	20.9/19.3	Day 5	Implantation Clinical pregnancy Ongoing pregnancy Miscarriage
Shapiro 2011b	137 (70/67)	33.0/32.9	12.9/14.1	Day 5	Implantation Clinical pregnancy Ongoing pregnancy Miscarriage
Chen 2016	1508 (746/762)	28.1/28.2	14.4/14.2	Day 2 Day 3 Day 5	Biochemical pregnancy Clinical pregnancy Ongoing pregnancy Miscarriage Live births
Vuong 2016	782 (391/391)	31.8/32.1	12.6/12.9	Day 3	Ongoing pregnancy Live birth
Coates 2017	179 (91/88)	36.6/36.7	14.0/17.0	Day 6 (PGS)	Implantation Ongoing pregnancy Live birth

### Systematic Review

Shapiro *et al.*, 2011a (High-responder): A prospective randomized trial was performed to assess potential effects of COS on endometrial receptivity. It was published, as correspondence, thus complete data on methods were not evaluated ("not-randomized", "not-blind", "no-power calculation" descriptions). Clinical pregnancy rates per transfers in Freeze/All-ET cycles and Fresh-ET were compared. The inclusion criteria were patients undergoing their first IVF cycle, day 3 FSH cycle <10IU/L, and >15 antral follicle-count. This study involved 131 patients, and 122 were randomized (62 to the fresh group and 60 to the cryopreservation group). The two groups were similar in age, antral follicle count, days of stimulation (10.4 versus 10.6), mean number of oocytes retrieved for Freeze/All-ET group (20.9±8.2) and Fresh-ET group (19.3±8.6), etc. The ongoing pregnancy rates per retrieval were 63.3% (38/60) in the Freeze/All-ET group and 54.8% (34/62) in the Fresh-ET group (*p*=0.36). Regression logistics was performed to check for potential differences in clinical outcomes while controlling for embryo quality. They found that a greater likelihood of clinical pregnancy was associated with the Freeze/All-ET group (*p*=0.0037).

Shapiro *et al.*, 2011b (Normo-responder): In this prospective randomized study of 137 patients undergoing their first IVF cycle in which they had 67 and 70 oocytes retrieved in the Fresh-ET and Freeze/All-ET groups, respectively, the authors compared success rates between Fresh-ET after ovarian stimulation and Freeze/All-ET after artificial endometrial preparation - to compare endometrial receptivity. A two-stage, two-sided group sequential procedure with an overall type I error of.05 was used, to test the primary hypothesis of a difference in the probabilities of clinical pregnancy for the two arms in this study, with a maximum sample size of 411 patients needed to achieve 80% power for detecting a difference of 15% in the clinical pregnancy rate (sample size not reached). Patients were randomized by drawing randomly among identical, opaque, unmarked sealed envelopes (there was no blind description). The two groups were similar in age, diagnosis, baseline serum FSH level, antral follicle count, days of stimulation (10.5 versus 10.4), mean number of oocytes retrieved (12.9±4.7 for Freeze/All-ET group and 14.1±6.4 for Fresh-ET group), etc. Both groups did not differ significantly in number of transferred blastocyst or endometrial thickness on the trigger day. There were no significantly greater rates of clinical pregnancy per randomized patient (60.0% versus 43.3%), ongoing pregnancy per randomized patient (55.7% versus 40.3%), and no significant lower miscarriage rate from clinical pregnancy (14.3% versus 24.1%) in the Freeze/All-ET group. Patients with extreme high responses were taken off the study.

Chen *et al*., 2016: assessed 1,508 infertile women with PCOS, who were randomized during their first IVF cycle to undergo either Fresh-ET (n=762) or Freeze/All-ET (n=746). The patients were randomly assigned to one of the two study groups in a 1:1 ratio, using an online central randomization system, which was unknown to the clinical investigators. Both groups had similar IVF cycle characteristics, including age, endometrial thickness, days of stimulation (10.3 versus 10.3) and number of oocytes retrieved (14.4±6.0 for the Freeze/All-ET group and 14.2±5.8 for the Fresh-ET group). They found that the Freeze/All-ET group achieved significantly higher live births rate (49.3% versus 42.0%); higher, but not significant, clinical pregnancy rates (58.7% versus 56.2%) and ongoing pregnancy rates (52.7% versus 48.8%). On the other hand, miscarriage rates (from clinical pregnancies) were significantly lower in the Freeze/All-ET group (14.6% versus 25.0%). The authors also compared perinatal outcomes.

Vuong *et al.*, 2016: In this randomized study, the aim was to compare the effectiveness of the Freeze/All-ET to conventional Fresh-ET in non-PCOS women. The inclusion criteria were: had ≤1 previous IVF cycle, could have embryo transfer on day 3, had at least 1 top-quality embryo. On the other hand, the exclusion criteria were: PCOS and oocyte donation. The days of stimulation were similar between the Freeze/All-ET and the Fresh-ET groups (9.16 versus 9.14). Randomization (1:1) was made by a computer-generated list. The sample size of 780 patients needed to achieve an 80% power for detecting a difference of 10% in ongoing pregnancy rates (there was no blind description). A total of 782 patients were included (391 in the Freeze/All-ET group and 391 in the Fresh-ET group). The primary outcome was ongoing pregnancy rates after the first embryo transfer. The baseline characteristics were similar between the groups, including age, stimulation duration and number of oocytes retrieved (12.6±5.6 for the cryopreservation group and 12.9±5.16 for the fresh group). They found no difference between the Freeze/All-ET and the Fresh-ET groups regarding ongoing pregnancy rates (36.3% versus 34.5%, respectively).

Coates *et al.*, 2017: In this clinical trial, the aim was to identify which embryo transfer strategy, after preimplantation genetic screening (PGS) by next generation sequencing (NGS), freeze-all or Fresh-ET, would improve clinical outcomes or whether the strategies were equally successful. Women between the ages of 18 and 42 years, while undergoing IVF and PGS using their own eggs, were eligible to participate in the trial. The exclusion criteria included a need to use surgically retrieved sperm, patients using preimplantation genetic diagnosis for a single-gene or chromosomal disorder, egg donor cycles, gender selection cycles, decreased ovarian reserve (early follicular phase serum FSH level >10IU/L or random serum anti-Mullerian hormone level <1ng/ml), and any medical conditions occurring before recruitment. A total of 179 patients were randomized to either a Freeze/All-ET cycle (91) or a Fresh-ET (88) on day 6 during the stimulated cycle. A professional third party prepared the stratified block randomization sequence. The allocation sequence was stratified for female age (<35, 35-37, 38-40, and 41-42 years) and number of prior ART cycles (≤2 or ≥3). The women were randomized in a 1:1 ratio. The two groups were similar in age, anti-Mullerian hormone levels, FSH levels, mean number of oocytes retrieved (17.0 for Freeze/All-ET group and 14.0 for Fresh-ET group), etc. Frozen ETs were performed in an artificial cycle, and Fresh-ET were carried out during original egg retrieval cycle. The outcome of patients in the intention-to-treat analysis were: ongoing pregnancy rates (40.9% vs*.* 62.2%; *p*<0.1) and live birth rates (39.8 vs*.* 61.5%; *p*<0.1) per intended treatment was significantly higher for the freeze-all group compared with the fresh group.

### Quality assessment

The methodological quality systems differ among the 5 RCTs. One trial did not have its complete data evaluated on methods ("non-randomized", "not-blind", "no-power calculation" descriptions) (Shapiro *et al.*, 2011a). Randomization was done by drawing randomly among identical, opaque, unmarked sealed envelopes in one study (Shapiro *et al.*, 2011b). In one study, the patients were randomly assigned to one of the two study groups in a 1:1 ratio, by an online central randomization system, which was unknown to the clinical investigators (Chen *et al.*, 2016). Drawing randomly (1:1) was made by a computer-generated list in one study (Vuong *et al.*, 2016). In one trial, a professional third party prepared the stratified block randomization sequence, and the allocation sequence was stratified for female age (Coates *et al.*, 2017). Two studies described the method of blinding (Chen *et al.*, 2016; Coates *et al.*, 2017).

### Main outcomes

The results of this meta-analysis were broken down into two parts, in accordance with the mean number of oocytes retrieved:

#### I- Outcomes when >12 to <21 oocytes retrieved

##### Clinical pregnancy rates (Figure 2)

**Figure 2.**
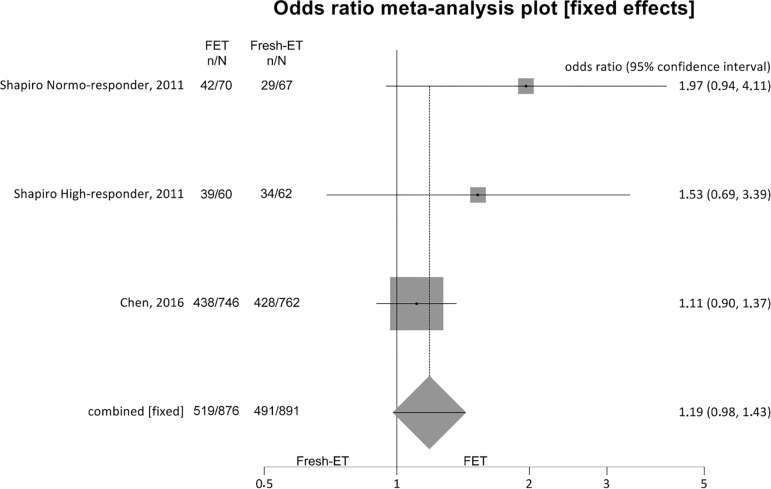
Clinical pregnancy rates when >12 and <21 oocytes were retrieved.

To analyze clinical pregnancy rates (per randomized patient), 3 studies were included, and there were no significant differences between the Fresh-ET group: 55.1%, (491/891) and the Freeze/All-ET group: 59.2% (519/876) (OR=1.19; 95%CI=0.98-1.43; *p*=0.09). There was no significant heterogeneity in this comparison: I^2^=33.2%; Cochran Q=2.99, *p*=0.22.

##### Ongoing pregnancy rates (Figure 3)

**Figure 3.**
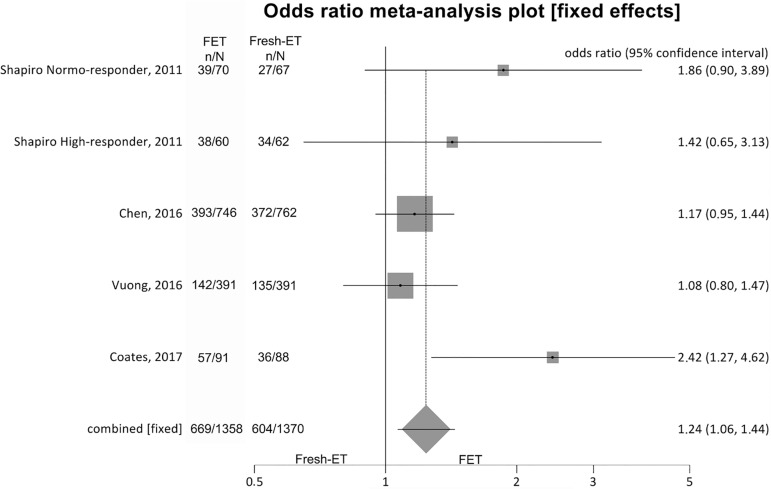
Ongoing pregnancy rates when >12 and <21 oocytes were retrieved.

To analyze ongoing pregnancy rates (per randomized patient), we included 5 studies and achieved significant differences between the groups: Fresh-ET: 44.1% (604/1370) versus Freeze/All-ET: 49.3% (669/1358) (OR=1.24; 95%CI=1.06-1.44; *p*=0.006). There was no significant heterogeneity in this comparison: I^2^=46.5%; Cochran Q=7.4, *p*=0.11.

##### Live birth rates (Figure 4)

**Figure 4.**
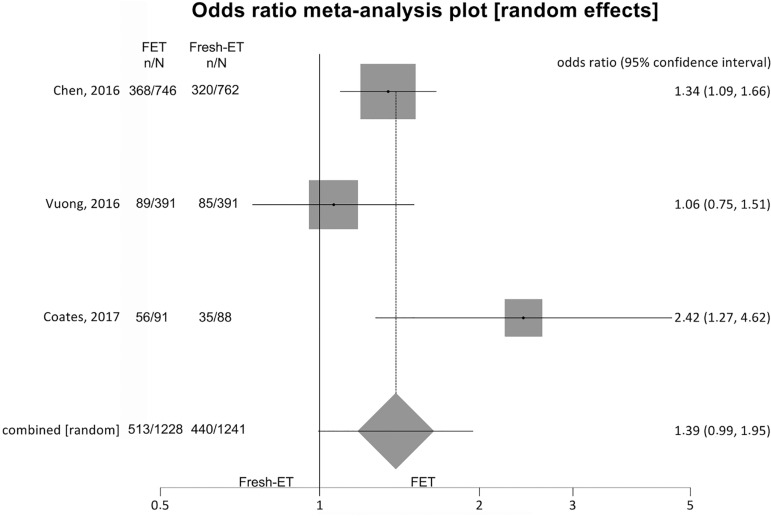
Live birth rates when >12 and <21 oocytes were retrieved.

To analyze live birth rates (per randomized woman) we included 3 trials and no significant difference was found between the groups: Fresh-ET: 35.5% (440/1241) versus Freeze/All-ET: 41.8% (513/1228) (OR=1.39; 95%CI=0.99-1.95; *p*=0.06). There was an important heterogeneity in this comparison: I^2^ =64.1%; Cochran Q=5.6; *p*=0.06.

##### Miscarriage rates

To analyze the rate of miscarriage (from clinical pregnancy), we considered 3 trials, and no significant difference was found between the groups: Fresh-ET: 13.8% (68/491) versus Freeze/All-ET: 10.0% (52/519) (OR=0.68; 95%CI=0.46-1.00; *p*=0.06). There was no heterogeneity in this comparison: I^2^=0%; Cochran Q=0.21, *p*=0.90.

#### II- Outcomes when >12 to <15 oocytes retrieved

##### Clinical pregnancy rates (Figure 5)

**Figure 5.**
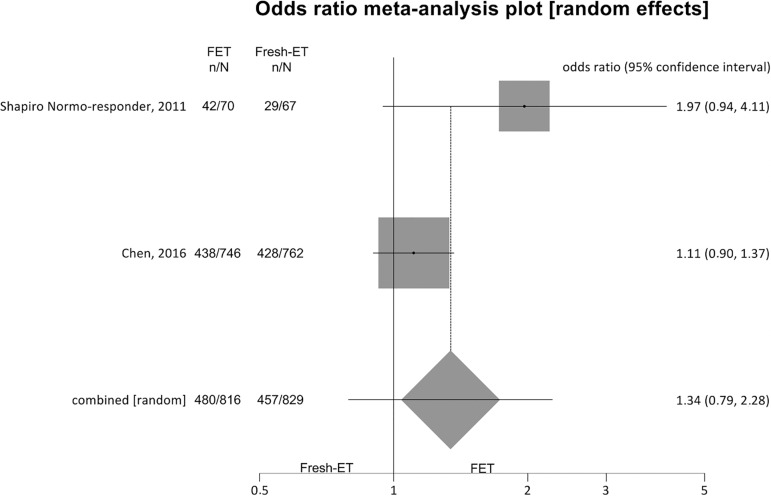
Clinical pregnancy rates when > 12 and < 15 oocytes were retrieved.

For clinical pregnancy rates (per randomized patient) we included 2 studies, and no significant difference was found between the fresh and the cryopreservation groups: Fresh-ET group: 55.1% (457/829) versus Freeze/All-ET group: 58.8% (480/816) (OR=1.34; 95%CI=0.79-2.28; *p*=0.27). The heterogeneity was measured: I^2^=NA; Cochran Q=2.5, *p*=0.11.

##### Ongoing pregnancy rates (Figure 6)

**Figure 6.**
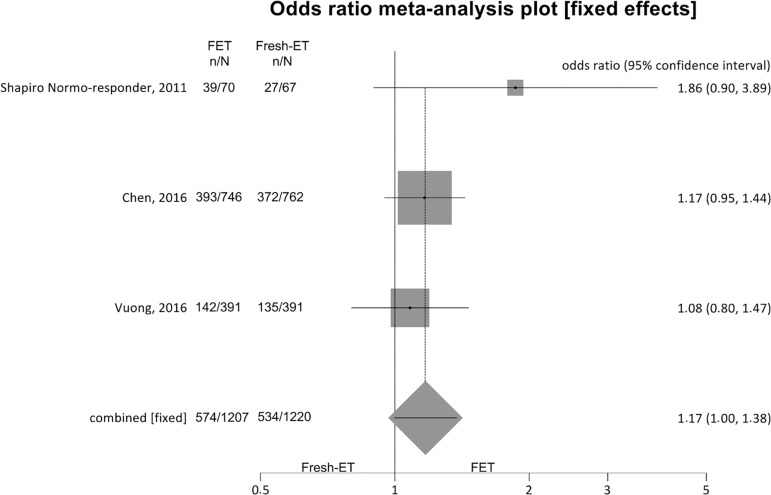
Ongoing pregnancy rates when >12 and <15 oocytes were retrieved.

For ongoing pregnancy rates (per randomized patient), we included 3 studies, and no significant difference was found between the groups: Fresh-ET group: 43.7% (534/1220) versus Freeze/All-ET group: 47.5% (574/1207) (OR=1.17; 95%CI=1.00-1.38; *p*=0.06). There was no significant heterogeneity in this comparison: I^2^=4.1%; Cochran Q=2.1, *p*=0.4.

##### Live birth rates (Figure 7)

**Figure 7.**
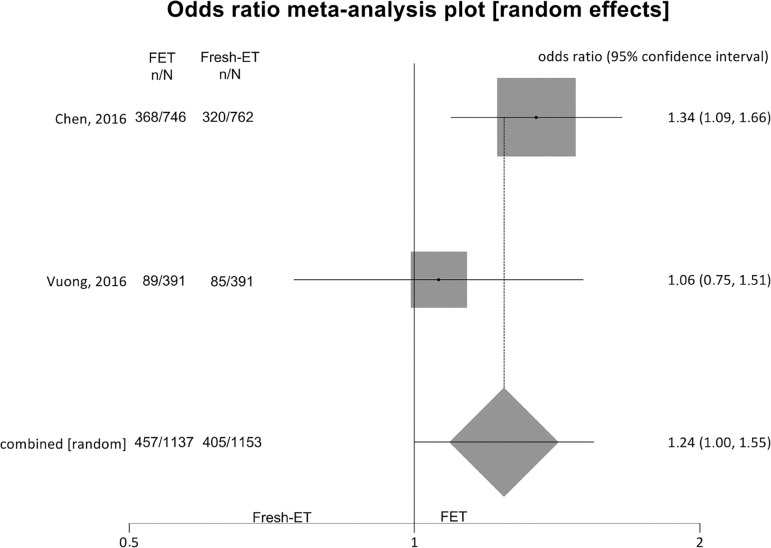
Live birth rates when >12 and <15 oocytes were retrieved.

To analyze live birth rates (per randomized woman) we included 2 studies, and no significant difference was found between the groups: Fresh-ET: 35.5% (405/1153) versus Freeze/All-ET: 41.8% (457/1137) (OR=1.24; 95%CI=1.00-1.55; *p*=0.05). The heterogeneity was measured: I^2^= NA; Cochran Q=1.4; *p*=0.2.

##### Miscarriage rates

For miscarriage rates (from clinical pregnancy) we included 3 studies, and no significant difference was found between the groups: Fresh-ET group: 13.8% (63/457) versus Freeze/All-ET group: 10.0% (48/480) (RR=0.68; 95%CI=0.46-1.02; *p*=0.06). The heterogeneity was measured: Cochran Q=0.2, *p*=0.65.

A summary of the results of the present meta-analysis comparing Freeze/All-ET and Fresh-ET strategies is depicted on Table 2, including all trials (when the mean number of oocytes collected was >12 and <21), and on Table 3, including trials with the mean number of oocytes retrieved between >12 and <15.

**Table 2:** Summary of the results: when the mean number of oocytes collected was >12 and <21

Outcome measured	No. of patients	Freeze/All-ET group	Fresh-ET group	Odds ratio	95% CI	Analysis model	Heterogeneity I^2^
Clinical pregnancy	1767	876	891	1.19	0.98-1.43	Fixed	33.2%
Ongoing pregnancy	2728	1358	1370	1.24	1.06-1.44	Fixed	46.5%
Live birth	2469	1228	1241	1.39	0.99-1.95	Random	64.1%
Miscarriage	1010	519	491	0.68	0.46-1.00	Fixed	0%

**Table 3:** Summary of the results: when the mean number of oocytes collected was > 12 and < 15

Outcome measured	No. of patients	Freeze/All-ET group	Fresh-ET group	Odds ratio	95% CI	Analysis model	Heterogeneity I^2^
Clinical pregnancy	1645	816	829	1.34	0.79-2.28	Random	NA
Ongoing pregnancy	2427	1207	1220	1.17	1.00-1.38	Fixed	4.1%
Live birth	2290	1137	1153	1.24	1.00-1.55	Random	NA
Miscarriage	937	480	457	0.68	0.46-1.02	Random	NA

### Publication bias

In the present meta-analysis concerning the freeze-all versus the fresh embryo transfers strategies, the publication biases were evaluated by Begg-Mazumdar (*p*=0.82), and Egger's tests (*p*=0.12). Visual inspection of Begg's funnel plots is available in the S1 Figure.

## DISCUSSION

When there are uncertainties about a given medical question, a meta-analysis is an important tool, able to dissolve such problem. This analytical method consists of an approach in which different and independent studies are joined and the results are combined into a single common outcome. Compared with narrative reviews, meta-analyses have the great advantage of being less influenced by a reviewer's opinion, thus providing unbiassed conclusions. In addition, all the results can easily be recalculated and compared with the conclusions stated by the authors. Regarding endometrial receptivity, several procedures are being proposed to improve clinical outcomes in patients undergoing ART cycles, and the freeze-all strategy seems to be an important step in this direction (Shapiro *et al.*, 2008; Shapiro *et al.*, 2011a, 2011b; Chen *et al.*, 2016; Vuong *et al.*, 2016; Coates *et al.*, 2017). This systematic review demonstrated that compared with Fresh-ET, the Freeze/All-ET brought about significant improvements to the ongoing pregnancy rates of patients submitted to ART procedures, when the mean number of oocytes collected was not limited to 15, regardless of having patients with PCOS. However, the Freeze/All-ET does not bear advantages when compared with Fresh-ET, when the mean number of oocytes retrieved is less than 15. These findings may be associated with the deleterious effects of COS on endometrial receptivity during ART cycles (Shapiro *et al.*, 2011a; Chen *et al.*, 2016).

There are several reasons that justify the employment of the freeze-all strategy, such as risk of ovarian hyperstimulation syndrome (OHSS), inadequate endometrial thickness, previous assisted reproduction procedure failures, infertility related to endometriosis, and high risk of venous thrombosis during ART procedures. However, the main pathophysiologic mechanism involved in the selection of the freeze-all strategy seems to be a premature progesterone elevation during COS, resulting in an impaired-reception uterine environment (Shapiro *et al.*, 2011b; Mohamed *et al.*, 2011; Nelson, 2013).

There is evidence in the literature to support this negative relationship between COS and pregnancy rates, probably due to the presence of elevated serum P and E levels during the follicular phase, promoting premature luteinization (PL), which occurrence is seen in up to 30% of IVF/ICSI cycles (Schoolcraft *et al.*, 1991; Fanchin *et al.*, 1993; Givens *et al.*, 1994; Venetis *et al.*, 2013). Possible explanations for PL occurrence, could be associated with the rising levels of E, that may induce increased LH secretion, able to stimulate granulosa cells to produce progesterone but unable to promote trigger ovulation (Ubaldi *et al.*, 1995; Melo *et al.*, 2006), and increases in the number of mature follicles with 17mm or more (Peluso, 1990; Bosch *et al.*, 2003; Glamočlija *et al.*, 2005). In addition, increased concentration of estrogen during the follicular phase in COS, upregulates endometrial progesterone receptor expression in comparison with what happens in natural cycles, promoting advanced endometrial maturation (Koo *et al.*, 2015). The success of ART cycles is dependent on the number and quality of oocytes and embryos, and endometrial receptivity (Schoolcraft *et al.*, 1991; Kagawa *et al.*, 1992; Silverberg *et al.*, 1994; Sims *et al.*, 1994; Bosch *et al.*, 2003; Lai *et al.*, 2009; Milachich & Shterev, 2016). The main negative effect of P elevation during ART procedures seems to be on endometrial receptivity (endometrial asynchrony), rather than on oocyte or embryo quality (Lu *et al.*, 2016). This harmful effect of P elevation on endometrial receptivity in patients undergoing fresh autologous IVF/ICSI cycles becomes more evident knowing that the highest pregnancy rates occur in fresh oocyte donation cycles, wherein the endometrium is artificially prepared, without deleterious COS effects (Legro *et al.*, 1993; Silverberg *et al.*, 1994; Shapiro *et al.*, 2009).

In view of the plausible negative effects of COS, mainly in high-responders, it has been demonstrated that the Freeze/All-ET could be the better choice to improve clinical outcomes in patients with higher P levels (Shapiro *et al.*, 2011a; Lu *et al.*, 2016). However, Levi and collaborators (in a non-randomized study) suggested that in patients submitted to ART procedures with a mean number of oocytes collected greater than 15, COS did not result in damage on endometrial receptivity, and the relative brief COS with reduced number of days of ovarian stimulation (8.4 days) could explain the reduced negative endometrial effect (Levi *et al.*, 2001).

On the other hand, the RCTs on freeze-all strategy available in the literature do not differ vis-à-vis outcomes involving ongoing pregnancy rates per randomized patient in the group of women with a mean number of retrieved oocytes below 15. Shapiro and collaborators demonstrated, in a prior RCT involving normal-responders, that ongoing pregnancy rates (per patient) was not higher in the group submitted to Freeze/All-ET, when compared with the group of patients in whom Fresh-ET was performed (Shapiro *et al.*, 2011b). Also, agreeing with the outcomes of this systematic review, Vuong and collaborators showed that patients with a mean number of collected oocytes of approximately 13, did not benefit from the Freeze/All-ET (Vuong *et al.*, 2016). Similarly, Chen and collaborators reported that the Freeze/All-ET group achieved higher, but not significant ongoing pregnancy rates (per patient) (Chen *et al.*, 2016).

A meta-analysis is a powerful tool, considered the highest in the evidence-based pyramid, but its strength depends on the quality of the randomized trials analyzed (Franco & Oliveira, 2015). Recently, an RCT including high-responders, favoring the Freeze/All-ET strategy was retracted of the literature access (Aflatoonian *et al.*, 2010). This retracted RCT is part of a relatively recent meta-analysis (Roque *et al.*, 2013) concerning the beneficial effects of cryopreservation and subsequent FET. However, removing the aforementioned study, the prior meta-analysis (Roque *et al.*, 2013) loses its power to assist in medical decision-making whether the Freeze/All-ET should be used or not in clinical practice.

The freeze-all strategy is a topic that has recently gained attention from clinicians and embryologists. However, although it has great relevance for advances in ART, more prospective and randomized trials, involving large populations are necessary to define whether delayed frozen-thawed embryo transfer is beneficial, and for which groups of patients it could provide improvements in the clinical outcomes of IVF/ICSI cycles.

In conclusion, the findings of this meta-analysis suggest that the freeze-all strategy could be favorable when high numbers of oocytes are collected, signaling an association between higher COS and consequent impairment in endometrial receptivity. However, when the mean number of oocytes collected is less than 15, the freeze-all strategy does not appear to be advantageous. More RCTs are required to evaluate whether the freeze-all strategy could influence clinical outcomes.

## Figures and Tables

**S1 File:** PRISMA Checklist

Section/topic	#	Checklist item
**TITLE**		
Title	1	Identify the report as a systematic review, meta-analysis, or both.
**ABSTRACT**		
Structured summary	2	Provide a structured summary including, as applicable: background; objectives; data sources; study eligibility criteria, participants, and interventions; study appraisal and synthesis methods; results; limitations; conclusions and implications of key findings; systematic review registration number.
**INTRODUCTION**		
Rationale	3	Describe the rationale for the review in the context of what is already known.
Objectives	4	Provide an explicit statement of questions being addressed with reference to participants, interventions, comparisons, outcomes, and study design (PICOS).
**METHODS**		
Protocol and registration	5	Indicate if a review protocol exists, if and where it can be accessed (e.g., Web address), and, if available, provide registration information including registration number.
Eligibility criteria	6	Specify study characteristics (e.g., PICOS, length of follow-up) and report characteristics (e.g., years considered, language, publication status) used as criteria for eligibility, giving rationale.
Information sources	7	Describe all information sources (e.g., databases with dates of coverage, contact with study authors to identify additional studies) in the search and date last searched.
Search	8	Present full electronic search strategy for at least one database, including any limits used, such that it could be repeated.
Study selection	9	State the process for selecting studies (i.e., screening, eligibility, included in systematic review, and, if applicable, included in the meta-analysis).
Data collection process	10	Describe method of data extraction from reports (e.g., piloted forms, independently, in duplicate) and any processes for obtaining and confirming data from investigators.
Data items	11	List and define all variables for which data were sought (e.g., PICOS, funding sources) and any assumptions and simplifications made.
Risk of bias in individual studies	12	Describe methods used for assessing risk of bias of individual studies (including specification of whether this was done at the study or outcome level), and how this information is to be used in any data synthesis.
Summary measures	13	State the principal summary measures (e.g., risk ratio, difference in means).
Synthesis of results	14	Describe the methods of handling data and combining results of studies, if done, including measures of consistency (e.g., I^2^) for each meta-analysis.
Risk of bias across studies	15	Specify any assessment of risk of bias that may affect the cumulative evidence (e.g., publication bias, selective reporting within studies).
Additional analyses	16	Describe methods of additional analyses (e.g., sensitivity or subgroup analyses, meta-regression), if done, indicating which were pre-specified.
**RESULTS**		
Study selection	17	Give numbers of studies screened, assessed for eligibility, and included in the review, with reasons for exclusions at each stage, ideally with a flow diagram.
Study characteristics	18	For each study, present characteristics for which data were extracted (e.g., study size, PICOS, follow-up period) and provide the citations.
Risk of bias within studies	19	Present data on risk of bias of each study and, if available, any outcome level assessment (see item 12).
Results of individual studies	20	For all outcomes considered (benefits or harms), present, for each study: (a) simple summary data for each intervention group (b) effect estimates and confidence intervals, ideally with a forest plot.
Synthesis of results	21	Present results of each meta-analysis done, including confidence intervals and measures of consistency.
Risk of bias across studies	22	Present results of any assessment of risk of bias across studies (see Item 15).
Additional analysis	23	Give results of additional analyses, if done (e.g., sensitivity or subgroup analyses, meta-regression [see Item 16]).
**DISCUSSION**		
Summary of evidence	24	Summarize the main findings including the strength of evidence for each main outcome; consider their relevance to key groups (e.g., healthcare providers, users, and policy makers).
Limitations	25	Discuss limitations at study and outcome level (e.g., risk of bias), and at review-level (e.g., incomplete retrieval of identified research, reporting bias).
Conclusions	26	Provide a general interpretation of the results in the context of other evidence, and implications for future research.
**FUNDING**		
Funding	27	Describe sources of funding for the systematic review and other support (e.g., supply of data); role of funders for the systematic review.

From: Moher et al., 2009. For more information, visit: www.prisma-statement.org

**S1 Figure.**
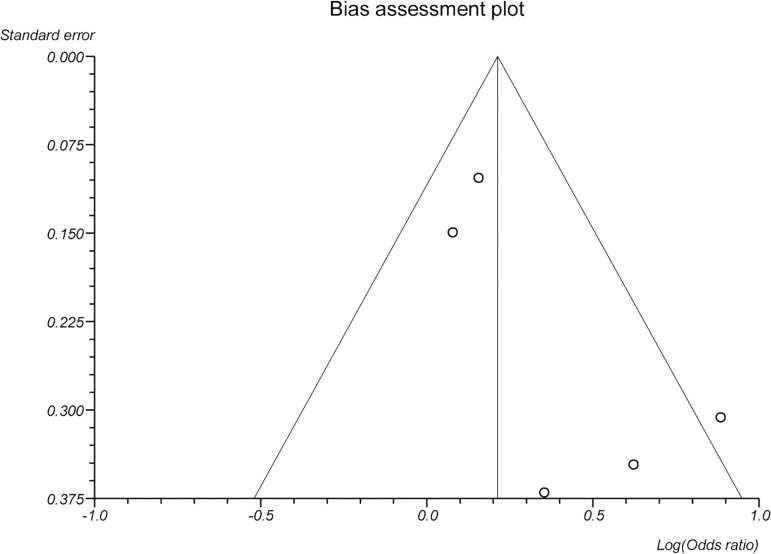
Beeg’s funnel plots (Publication Bias).
